# Royal Sea-Bathing Infirmary at Margate

**Published:** 1893-02-25

**Authors:** 


### HOSPITAL MEETINGS.

### ROYAL SEA-BATHING INFIRMARY AT MARGATE

ON the 16th inst. a meeting in aid of the Royal Sea-bathing Infirmary at Margate was held at Lambeth Palace. The beautiful library was crowded, and the speeches were listened to with marked attention and interest. The Archbishop of Canterbury presided, and Viscount Cranbrook, Sir Trevor Lawrence, Sir James Paget, Sir Andrew Clark, Mr. Bryant, Mr. Jonathan Hutchinson, Mr. Treves, and Mr. Brudenell Carter were amongst the speakers. Seldom indeed has so much eloquence graced a single meeting, and many were the gratulatory remarks exchanged by those whose good fortune it was to be present on an occasion which will not easily be forgotten.

The infirmary may be called a national one; it was built in 1791 not for Margate, but "in the first place for London, and then for the whole country." It only accommodated 16 patients when first opened; in 1881 the available beds numbered 250, but the hygienic arrangements were defective. Sir Erasmus Wilson spent £30,000 on the hospital, and the dangers of overcrowding were obviated; but, unhappily, the institution met with no increased support, and the annual deficit soon averaged £3,000. These deficiencies were met by successive sales of capital, and the governors and directors, being determined to keep out of debt, were finally driven to take the extreme step of closing 140 beds at the end of last year, thus reducing the number occupied to 80. It was then decided to try, by the present appeal to the public, to rouse general sympathy in an institution which should command universal support.

Viscount Cranbrook spoke of the charity being comparatively unknown, because "out of sight," and not given to advertising its wants. He spoke warmly of the Margate air, alluding pleasantly to the "witnesses of unimpeachable character" who would shortly give evidence, from a professional standpoint, as to its virtues. He thought that the fashionable "leaflet" might do good service to the charity if it merely embodied a list of the names of the many distinguished physicians and surgeons who gave evidence in its favour. He asked for continuous, not spasmodic, support.

Sir Trevor Lawrence, as a governor of the infirmary, offered figures and facts, and Sir James Paget spoke at length as to the need for placing the sick poor under as happy circumstances as the rich. In hospitals this was certainly the case, for with resident medical men, perpetual watching, excellent nursing and sanitary arrangements, the scrofulous poor had advantages which only the greatest wealth could secure for the tuberculous rich. The latter could only have long voyages, seaside or high Alps after all, at a great expense, while the poor enjoyed equal benefits at the Margate Infirmary. Sir James Paget and subsequent speakers dwelt on the curable nature of scrofula, and the steady progress to perfect recovery made in sea air. The patients became healthy persons, fit to maintain themselves and their offspring, and not merely invalids.

Sir Andrew Clark said, after careful investigations, he was convinced that this infirmary had greater claims on us than any other institution, and it was excellently and economically managed, but most inadequately supported. Its empty beds were a shame to England. Money was asked, not for building, but simply to maintain beds now empty. Certainly fifty or sixty years ago there was more "giving" than now, and "times" being "bad" was no valid excuse—rather the contrary, for when giving demanded a sacrifice it became divine. Margate Hospital being an old and tried servant, it merited help to enable it to continue its splendid work of restoring health and preserving lives.

Mr. Bryant spoke of the infirmary as a "unique charity," and specially called attention to the fact that when patients were able to remain at Margate a minor operation frequently sufficed, whereas in an inland hospital a major one would have been required. He called attention to the fact that, as each individual was the centre of a large circle, so it was his plain duty to interest each member of it in this matter. Himself, he considered the closing of wards at Margate as a public calamity.

Mr. Jonathan Hutchinson remarked on his pleasure at seeing the presidents of both colleges, and said he was strongly tempted to give his hearers a clinical lecture. He remarked "I should like to take you to the wards," referring to popular errors with regard to the clinical aspect of scrofula. He himself defined it as "the vigor of life being a little below par and distinctly not specific." Therefore patients needed invigoration, &c. The statements of so well known an authority were listened to with great interest by the many doctors present as well as by his unprofessional hearers. Mr. Hutchinson spoke of the old surgeon who defined scrofula as bad flesh to heal, and said "he was near the truth." He himself thoroughly agreed with Mr. Bryant that patients could get perfectly well and indeed were well worth curing as they were "cured once for all." Mr. Treves, senior surgeon to the infirmary, alluded to the situation of the institution, and explained that, being on the Isle of Thanet, it had the benefit of sea air in three out of the four quarters of the winds, and he praised the improved sanitation and present excellent nursing at the infirmary. The proceedings concluded with a vote of thanks to the Archbishop, who said he deserved no thanks, but considered it a great privilege to lend the room for such an occasion.

Donations given during the meeting, amounting to over £300, were announced by the Secretary.

